# Editorial of Special Issue “Frontiers in the Actin Cytoskeleton”

**DOI:** 10.3390/ijms21113945

**Published:** 2020-05-30

**Authors:** Francisco Rivero

**Affiliations:** Centre for Cardiovascular and Metabolic Disease, Hull York Medical School, Faculty of Health Sciences, University of Hull, Hull HU6 7RX, UK; Francisco.rivero@hyms.ac.uk; Tel.: +44-01482-466433

The actin cytoskeleton is of fundamental importance for eukaryotic cell homeostasis. It contributes to developing and maintaining cell shape and tissue integrity and is crucial for cell migration, movement of organelles, vesicle trafficking, and the completion of cell division. Impressive advances have been made in recent years towards understanding the intricacies of the microfilament system’s organization and function. This Special Issue of *IJMS* covers a broad range of cutting-edge aspects related to the actin cytoskeleton.

The mechanical properties of the cell are intimately linked to the highly dynamic and, at the same time, highly crosslinked cytoskeletal structures that occupy the cytoplasm. Liu et al. [1] use atomic force microscopy indentation coupled to image recognition-based cytoskeleton quantification to quantify the effect of F-actin and microtubule morphology, achieved by various levels of depolymerization, on cellular mechanical properties, and conclude that living cells are able to sense and adapt to the polymerization state of their cytoskeleton components. Dozens of actin-binding proteins (ABPs) orchestrate the dynamic remodeling of the actin cytoskeleton and integrate it with the cell signaling machinery. ABPs have unique localizations within the crowded cytoplasm, in which they diffuse within seconds. How does a specific ABP find where to bind when the cytoplasm offers endless possibilities? Tokuraku et al. [2] discuss the concept of allosteric regulation of ABP localization that arises from cooperative conformational changes propagating along actin filaments upon binding of ABPs like myosin, tropomyosin, cofilin, and others. This phenomenon is proposed to play an important role in the formation and regulation of actin structures like stress fibers, lamellipodia, and filopodia. It probably also applies to more elaborate structures like the inner ear hair cell stereocilia. Hair cells are the specialized neuroepithelial cells responsible for detecting sound and head movements. Each of these cells carries an apical bundle of stereocilia that upon deflection activate ion channels, causing depolarization, neurotransmitter release, and excitation of auditory or vestibular nerves. Pacentine et al. [3] review the current knowledge on the morphology, composition, and role of the rootlet, a specialized structure that anchors the actin core of the stereocilium to the cell body.

Actin and ABPs play roles not only in structures built in the cytoplasm but also in extracellular vesicles released by the cells and used for intercellular communication. Holliday et al. [4] review the actin and actin-associated proteome of extracellular vesicles released by osteoclasts. They report the presence of members of several of the major classes of ABPs, suggesting that they are important for the formation of extracellular vesicles and for their regulatory function on osteoblasts. The power of proteomics is also showcased in the study of Fabrice et al. [5]. Here, the authors describe the interactome of the *Dictyostelium discoideum* amoeba coronin A. Coronins are evolutionary conserved cytoskeleton remodeling proteins, but actin-independent roles, particularly in signaling, are emerging. The study found coronin A in complex with a number of ABPs, but also several uncharacterized proteins, metabolic enzymes, and a transcription factor that will provide fodder for future studies. Co-sedimentation experiments in this study also put into question the relevance of the interaction of coronin A with actin, suggesting that the phenotypes observed in coronin A-deficient amoebae are mainly the result of altered signaling. In mammalian cells, one of the interaction partners of coronin 1 is the cytoplasmic tail of integrin β2, a component of lymphocyte-associated antigen 1, the fourth most abundant integrin in platelets. Coronins 1, 2, and 3 are abundant in platelets, but their roles are poorly understood. Riley et al. [6] describe the characterization of mouse platelets deficient in coronin 1 and show that the protein is dispensable for most cellular processes, most likely due to functional overlap among coronins, but is required for translocation of integrin β2 to the platelet surface upon stimulation with thrombin.

Nucleation-promoting factors activate the Arp2/3 complex to trigger actin nucleation. Several of those factors have been extensively studied over the last few decades, including the Wave complex. This complex is itself activated by interaction with activated small GTPases like Rac1. Singh et al. [7] investigate Arf6, a member of the ADP ribosylation factor family involved in a wide array of cellular functions. Some members of the Arf family, like Arf5 and Arl1, cooperate with Rac1 to recruit the Wave complex. In their study, Singh et al. now show that another Arf family member, Arf6, is not only capable of activating the Wave complex indirectly by recruiting the exchange factor ARNO, but also can trigger actin assembly directly in coordination with Rac1. Formins constitute another family of evolutionarily conserved cytoskeleton nucleators. Their hallmark is the FH2 domain that promotes the nucleation and elongation of linear actin filaments but can also associate to microtubules. Formins usually make for large families and in *Arabidopsis thaliana* the family has 21 members. While formins are known to form homodimers through their FH2 domain, heterodimerization has been seldom reported. Kollárová et al. [8] investigate two previously uncharacterized plant formins, AtFH13 and AtFH14, and although they show distinct and only partially overlapping patterns of subcellular localization, they are capable of heterodimerizing, a finding not reported previously in plant formins. As important as nucleation in the process of actin remodeling are mechanisms like severing, depolymerization, and regeneration of actin monomers, to which cyclase associated proteins (CAPs) contribute in complex ways. Two isoforms of CAP exist in mammalian cells, but while CAP1 has been extensively studied biochemically, CAP2 has never been. Purde et al. [9] show in their study that the N-terminal domain of both isoforms enhances cofilin-mediated severing and depolymerization of actin filaments. By studying the association status of CAPs, the authors noted that these activities are directly proportional to the degree of oligomerization, with monomers being less effective than tetramers.

About one hundred ABPs contribute to organize the cytoskeleton at the cell cortex, a dense meshwork associated with the plasma membrane. This cortical network is important for the generation of tension needed to maintain cell shape and polarity and to make cell motility possible. Ezrin, radixin, and moesin proteins are among the ABPs that regulate the organization of cortical actin filaments. García-Ortiz and Serrador [10] review the main biochemical mechanisms involved in the regulation of members of this family and their contribution to leukocyte biology, with a focus on the phagocytic cup and the immune synapse. A particular example of the roles of the cortical actin cytoskeleton is the first division of the *Caenorhabditis elegans* embryo, a model of asymmetric cell division that integrates microfilaments, microtubules, and complex signal cues. Samandar Eweis and Plastino [11] review recent research on the roles of the actin cytoskeleton in this crucial stage of the morphogenesis of the worm embryo, with a focus on the processes of symmetry breaking, cortical flows that help establish polarity, and contractile ring formation and positioning.

Having fundamental roles in a plethora of cellular processes, it comes to no surprise that defects in actin and associated proteins have been found to be associated with various diseases. Humans express six actin genes, some of them in a tissue-specific manner, giving rise to highly similar proteins. Disease-causing mutations have been reported for each of the six genes. The most common mutations result in conditions like nemaline myopathy, aortic aneurysms, and cardiomyopathy. Parker et al. [12] review the mutations reported in the human actin genes, their potential consequences for actin function, and the challenges that actins pose for experimental studies. Actin is the major cytoskeletal component of dendritic spines, small protrusions along dendrites, which in the mammalian brain harbor the postsynaptic compartment of glutamatergic excitatory synapses. The actin cytoskeleton contributes decisively to maintaining the dendritic spine architecture and modulating its remodeling. Synaptic dysfunction driven by amyloid β is characteristic of the neurodegenerative disorder Alzheimer’s disease. Pelucchi et al. [13] review the role of the actin cytoskeleton in the spine shaping, the participation of actin and actin remodeling proteins in the endocytosis mechanisms implicated in amyloid generation and receptor trafficking, and the evidence supporting the implication of the actin cytoskeleton in synaptic failure.

In the skeletal muscle cell, the transmembrane protein dysferlin facilitates calcium-dependent aggregation and fusion of vesicles during repair of the plasma membrane, at which point it interacts with proteins involved in actin remodeling. Mutations in dysferlin cause a group of muscular dystrophies called dysferlinopathies. Báez-Matus et al. [14] investigate the potential effects of alterations in dysferlin expression on actin dynamics, more specifically G-actin incorporation to filaments. They use immortalized myoblast cell lines derived from dysferlinopathy patients or normal myoblasts in which the dysferlin gene has been silenced and conclude that dysferlin is important for the regulation of actin remodeling.

Many bacterial pathogens have developed the ability to manipulate the actin remodeling machinery to facilitate their own uptake by the host cell and subsequent proliferation and invasion of other cells within the organism. A particular example is the obligate intracellular bacterium *Chlamydia trachomatis*. This organism uses aspects of actin remodeling to induce its own uptake by the host epithelial cell, to create a replicative niche, and, in some cases, to promote its egress from the infected cell, as discussed by Caven and Carabeo [15] in their review.

Overall, the 15 contributions that make up this Special Issue highlight the fundamental roles of the actin cytoskeleton in cellular processes relevant to health and disease. The combination of molecular genetics, biophysics, and advanced imaging techniques in a variety of cell types and model organisms will ensure that exciting discoveries will continue to be made in this field in years to come.
